# Reviewing the Effects of l-Leucine Supplementation in the Regulation of Food Intake, Energy Balance, and Glucose Homeostasis

**DOI:** 10.3390/nu7053914

**Published:** 2015-05-22

**Authors:** João A.B. Pedroso, Thais T. Zampieri, Jose Donato

**Affiliations:** Department of Physiology and Biophysics, Institute of Biomedical Sciences, University of São Paulo, São Paulo 05508-000, Brazil; E-Mails: nutri.pedroso@gmail.com (J.A.B.P.); thaizampieri@gmail.com (T.T.Z.)

**Keywords:** branched-chain amino acids, obesity, diabetes mellitus, protein synthesis, central nervous system, mTOR

## Abstract

Leucine is a well-known activator of the mammalian target of rapamycin (mTOR). Because mTOR signaling regulates several aspects of metabolism, the potential of leucine as a dietary supplement for treating obesity and diabetes mellitus has been investigated. The objective of the present review was to summarize and discuss the available evidence regarding the mechanisms and the effects of leucine supplementation on the regulation of food intake, energy balance, and glucose homeostasis. Based on the available evidence, we conclude that although central leucine injection decreases food intake, this effect is not well reproduced when leucine is provided as a dietary supplement. Consequently, no robust evidence indicates that oral leucine supplementation significantly affects food intake, although several studies have shown that leucine supplementation may help to decrease body adiposity in specific conditions. However, more studies are necessary to assess the effects of leucine supplementation in already-obese subjects. Finally, although several studies have found that leucine supplementation improves glucose homeostasis, the underlying mechanisms involved in these potential beneficial effects remain unknown and may be partially dependent on weight loss.

## 1. Introduction

Several nutrients have nutritional properties that exceed their roles as energy sources or molecule precursors. This is the case for the branched-chain amino acid (BCAA) l-leucine (in this manuscript, we will use the term leucine). Leucine is an essential amino acid for protein synthesis. Additionally, similarly to other amino acids, the carbon skeleton of leucine can be used to generate ATP. However, leucine can also regulate several cellular processes such as protein synthesis, tissue regeneration, and metabolism. Therefore, leucine supplementation has been studied in a variety of conditions such as aging, muscle lesions, protein/energy deprivation, obesity, and diabetes mellitus. Because leucine availability influences signaling pathways involved in the regulation of metabolism and because the incidence of metabolic diseases has reached alarming levels worldwide, investigating nutritional supplements that are potentially beneficial for the treatment and prevention of obesity and diabetes mellitus has become of paramount importance. Thus, the objective of the present review was to summarize and discuss the available evidence regarding the mechanisms and the effects of leucine supplementation in the regulation of food intake, energy balance, and glucose homeostasis.

## 2. Intracellular Mechanisms Activated by Leucine

**Figure 1:**
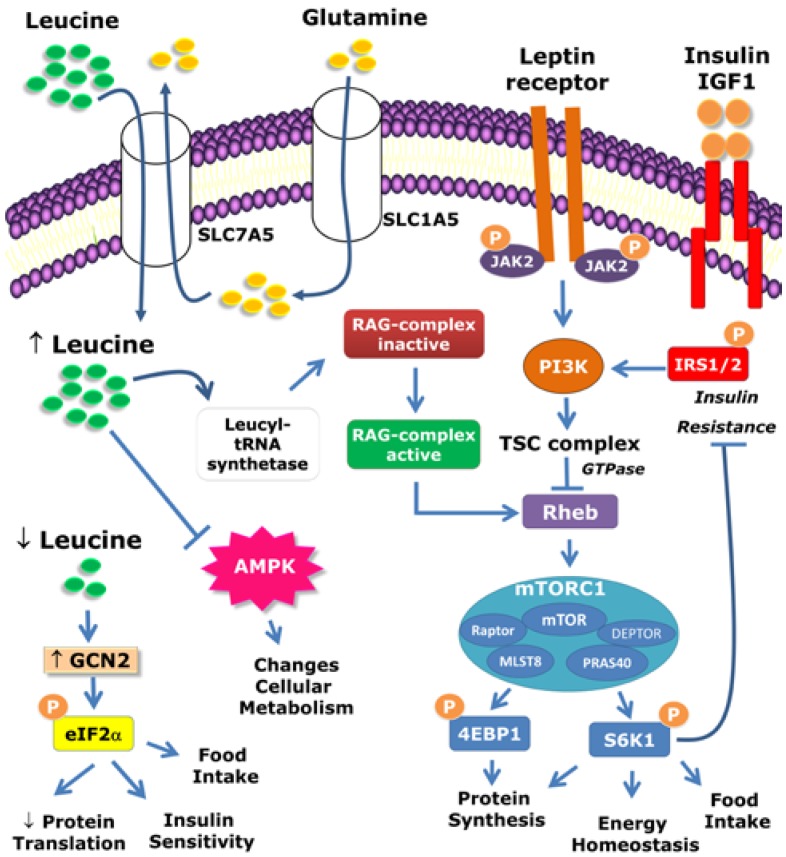
Intracellular mechanisms activated by leucine. The mammalian target of rapamycin complex 1 (mTORC1) comprises mTOR, Raptor, mLST8, PRAS40, and DEPTOR. mTORC1 is activated by amino acids (especially leucine) as well as by hormones such as leptin, insulin, and IGF-1. mTORC1 can be activated by different pathways. Hormonal activation primarily occurs through the TSC complex. However, amino acid-dependent mTORC1 activation occurs through the Rag complex. The leucyl-tRNA synthetase is responsible for sensing leucine cellular levels and activating the Rag complex. The cellular uptake of L-glutamine and its subsequent rapid efflux in the presence of leucine represent the rate-limiting step of mTOR activation. The protein p70-S6 kinase 1 (S6K1) and eukaryotic initiation factor 4E (eIF4E) binding protein 1 (4E-BP1) are key downstream targets of mTORC1. S6K1 also phosphorylates components of the insulin signaling pathway, which may lead to insulin resistance in situations of nutrient abundance such as in obesity. The anorexigenic effects of leptin require both the phosphatidylinositol-4,5-bisphosphate 3-kinase (PI3K) and mTOR/S6K1 signaling pathways. Because mTOR is a downstream target of PI3K signaling, the acute anorexigenic effects of leptin may depend on the PI3K/mTOR/S6K1 pathway.

For decades, amino acids have been known to be important regulators of protein synthesis [1]. Although protein synthesis can be stimulated by several isolated amino acids [2], leucine has a particularly potent effect [3,4,5,6]. The initiation of mRNA translation is the major mechanism by which leucine stimulates protein synthesis. Classical studies have shown that the regulation of mRNA translation by leucine is dependent on the mammalian target of rapamycin (mTOR) because rapamycin, a specific mTOR inhibitor, is able to blunt the effects of leucine [4,7,8,9]. mTOR is a serine/threonine kinase that is involved in the regulation of multiple cellular processes, including protein synthesis and cell growth, proliferation, and survival. mTOR controls protein synthesis through mTOR complex 1 (mTORC1), which comprises mTOR itself and other proteins, as follows: regulatory-associated protein of mTOR (Raptor), mammalian lethal with SEC13 protein 8 (MLST8), proline-rich Akt/PKB substrate 40 kDa (PRAS40), and DEP domain-containing mTOR-interacting protein (DEPTOR) (Figure 1). This protein complex is an important nutrient sensor that is regulated by amino acids (especially by leucine) as well as by growth factors and hormones that are secreted in response to nutrient ingestion (*i.e.*, insulin). mTORC1 has two major mechanisms of activation, via the tuberous sclerosis complex (TSC1/2) and the Rag complex. Hormones such as insulin and insulin growth factor-1 (IGF-1) activate the mTORC1 complex primarily through the TSC complex [10]. However, amino acid-dependent mTORC1 activation occurs through the Rag complex [11] (Figure 1). The presence of amino acids activates Rag GTPase heterodimers, which interact with Raptor. This interaction changes the intracellular localization of mTOR to a compartment that also contains the Ras homolog enriched in brain (Rheb) protein, which leads to mTORC1 complex activation [11]. Recent studies have shown that the enzyme that catalyzes the ligation of leucine to its transfer RNA (tRNA) is responsible for sensing leucine cellular levels and activating the Rag complex [12]. In this regard, leucyl-tRNA synthetase plays a non-canonical role by directly binding to Rag GTPase in an amino acid-dependent manner, and acts as a GTPase-activating protein for Rag GTPase to promote mTORC1 activation [12] (Figure 1). Additionally, the cellular uptake of L-glutamine and its subsequent rapid efflux in the presence of essential amino acids (*i.e.*, leucine) represent a rate-limiting step in mTOR activation [13]. Blocking solute carrier family 1 member 5 (SLC1A5), which is a high-affinity L-glutamine transporter, leads to mTORC1 inhibition (Figure 1). mTORC1 activity depends on a bidirectional transporter that regulates the simultaneous efflux of L-glutamine out of cells and the transport of leucine (and other essential amino acids) into cells. This bidirectional amino acid transport is mediated by the heterodimeric, bidirectional antiporter solute carrier family 7 member 5 (SLC7A5)/SLC3A2 [13]. Overall, this system regulates the intracellular concentration of essential amino acids, which is required for Rag-mTORC1 complex activation (Figure 1).

The proteins p70-S6 kinase 1 (S6K1) and eukaryotic initiation factor 4E (eIF4E) binding protein 1 (4E-BP1) are key downstream targets of mTORC1 and are therefore influenced by leucine availability (Figure 1). These proteins, when phosphorylated by mTORC1, lead to mRNA translation initiation and protein synthesis. Although the S6 ribosomal protein is the classical target of S6K1 serine/threonine phosphorylation, S6K1 can also phosphorylate insulin signaling pathway components [14] (Figure 1). This effect is relevant to inducing insulin resistance in situations of nutrient abundance such as in obesity [14,15]. Hypothalamic S6K1 also regulates the energy balance [16]. These topics will be discussed later in this review. Leucine may also signal through other pathways in addition to the mTOR pathway. For example, several studies have indicated that leucine can modify AMP-activated protein kinase (AMPK) activation [17,18,19,20,21,22,23]. Leucine-induced modification of AMPK signaling possibly causes changes in cellular metabolism and may mediate some of the effects of leucine. Furthermore, the general control non-depressible kinase 2 (GCN2) pathway is also affected by leucine availability [24]. More specifically, leucine deprivation increases GCN2 signaling, which in turn phosphorylates eukaryotic initiation factor 2α (eIF2α). This effect leads to repression of protein translation [25]. The activation of this pathway by changes in leucine levels may alter insulin sensitivity [24]. Hypothalamic eIF2α signaling also regulates food intake [26].

## 3. Leucine-Responsive Tissues

**Figure 2:**
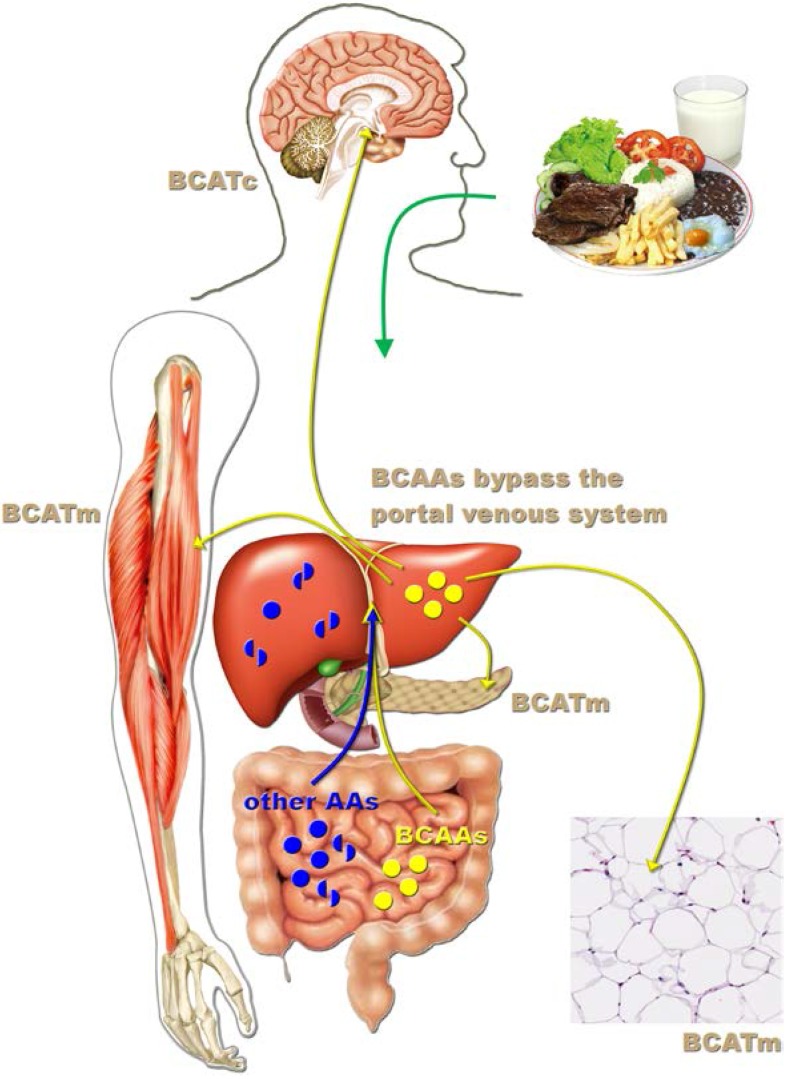
Leucine-responsive tissues. After protein-rich meals, circulating BCAA levels significantly increase, whereas other amino acids are highly metabolized by the gut or liver before reaching the systemic circulation. Branched-chain amino acid transaminase (BCAT) catalyzes the first and reversible transamination step of leucine degradation. This enzyme is not expressed in the liver, which allows the BCAAs to bypass the portal venous system following their intestinal absorption. In the brain, leucine is metabolized by the cytosolic form of BCAT (BCATc), whereas in other tissues (e.g., white adipose tissue, skeletal muscle, and pancreas), the mitochondrial form of BCAT (BCATm) prevails.

Previous studies have demonstrated that numerous tissues respond to acute and chronic leucine treatment (Figure 2). For example, oral leucine administration increases protein synthesis in the white adipose tissue, skeletal muscle, liver, heart, kidney, and pancreas [5,7,9,27,28,29,30]. In all of these tissues except the kidneys, leucine increases S6K1 and 4E-BP1 phosphorylation, indicating mTOR signaling pathway activation [9,28]. Oral leucine supplementation has also been shown to induce S6K1 phosphorylation in the hypothalamus [31]. Leucine metabolism depends on the first and reversible transamination step, which is catalyzed by the branched-chain amino acid transaminase (BCAT) enzyme (Figure 2). BCAT has two isoforms that are codified by different genes. The cytosolic form of BCAT (BCATc; encoded by the *Bcat1* gene) is highly expressed in the brain/peripheral nerves and is nearly absent in other tissues. The mitochondrial form of BCAT (BCATm; encoded by the *Bcat2* gene) is expressed in multiple tissues [27,32,33,34]. Importantly, neither isoform is expressed in the liver or gut, which allows the BCAAs to bypass the portal venous system without being metabolized following their intestinal absorption (Figure 2). This characteristic is unique among amino acids. Therefore, the systemic BCAA levels rise significantly after meals, allowing all of the tissues in the body to sense BCAA intake, whereas other amino acids are highly metabolized by the gut or liver before reaching the systemic circulation (Figure 2). The second and irreversible step in the metabolism of leucine is catalyzed by the branched-chain α-ketoacid dehydrogenase complex (BCKDK), which is expressed in numerous tissues. Previous studies have shown that BCAA oxidation is self-regulated. Consequently, increased BCAA levels induce higher BCAT and BCKDK complex activities [35,36]. This mechanism prevents excessive BCAA levels that otherwise could be toxic [37]. Therefore, changes in BCAT and BCKDK complex expression in response to leucine supplementation may represent an alternative way to identify leucine-responsive tissues. Zampieri *et al.* [31,38] found that chronic leucine supplementation in the drinking water increased BCATc, BCATm, and BCKDK expression in the hypothalamus of mice and rats consuming either a regular rodent diet or a high-fat diet (HFD). These results provided additional evidence that the central nervous system (CNS), including the hypothalamus, is also sensitive to changes in leucine intake.

## 4. Central Effects of Leucine

The CNS is an important site for regulating food intake, energy balance and glucose homeostasis [39]. Because leucine influences critical cellular processes through mTOR activation, it is important to determine whether this enzyme is expressed in the brain and exerts relevant effects in the nervous system. In this regard, Cota *et al.* [40] found that although mTOR is ubiquitously distributed in the CNS, the phosphorylated form of mTOR at Ser^2448^ (pmTOR) is more restrictively expressed. pmTOR is highly localized in the hypothalamic nuclei, which are involved in the regulation of energy balance, including the paraventricular nucleus of the hypothalamus (PVH) and the arcuate nucleus of the hypothalamus (ARH). A similar distribution pattern was observed for the activated form of S6K1 (phosphorylated at Thr^389^, pS6K1). Neurochemically and functionally defined populations of neurons exist in the ARH. Cells closer to the third ventricle co-express neuropeptide Y (NPY), agouti-related peptide (AgRP), and γ-aminobutyric acid (GABA). These cells stimulate food intake and are therefore inhibited by nutrient ingestion. Another population of cells located more laterally in the ARH co-expresses proopiomelanocortin (POMC) and cocaine- and amphetamine-regulated transcript (CART). These cells promote reduction in food intake and are activated by nutrient ingestion [39]. Approximately 90% of NPY/AgRP/GABA cells express pmTOR and pS6K1, whereas these phosphorylated proteins are found in 45% of POMC/CART cells in the ARH [40]. Fasting reduces hypothalamic pS6K1 and pmTOR expression [40]. Interestingly, intracerebroventricular (icv) leucine administration acutely decreases the food intake and body weight of rats, and these effects are blocked by rapamycin [40]. Another study found that changes in hypothalamic S6K1 activity modify the energy homeostasis of rats [16]. Virus-induced S6K1 hyper-activation in the mediobasal hypothalamus (MBH), which includes the ARH and other nuclei, decreased the NPY/AgRP expression, food intake, weight gain, and energy expenditure of rats [16]. Moreover, constitutive S6K1 activation in the MBH increases the acute anorexigenic effects of leptin and protects animals against diet-induced obesity and insulin resistance [16]. Additionally, icv infusion of the anorexigenic hormone leptin increases hypothalamic pS6K1 expression and reduces food intake and body weight in a rapamycin-dependent manner [16,40]. Overall, these results suggest that hypothalamic mTOR/S6K1 signaling regulates food intake and energy balance and mediates the acute anorexigenic effects of leptin. Because previous studies have suggested that the acute anorexigenic effects of leptin are also mediated by PI3K signaling [41,42] and that mTOR is a downstream target of PI3K signaling [10], the acute anorexigenic effects of leptin may depend on the PI3K/mTOR/S6K1 pathway (Figure 1).

**Figure 3:**
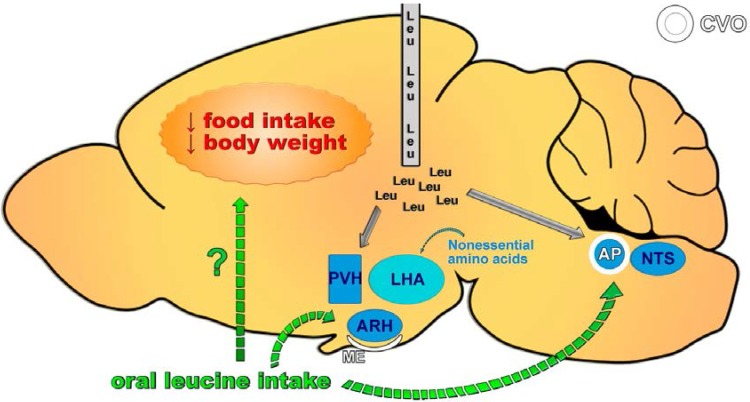
Neuronal circuitries required for the central effects of leucine on feeding. Central leucine administration (intracerebroventricular or parenchymal) acutely decreases food intake and body weight. This response is due to the activation of hypothalamic nuclei involved in regulating energy balance, including the paraventricular nucleus of the hypothalamus (PVH) and the arcuate nucleus of the hypothalamus (ARH), as well as extra-hypothalamic sites such as the nucleus of the solitary tract (NTS). Conversely, oral leucine administration does not induce neuronal activation in the PVH, ARH, or NTS but does cause c-Fos expression in the area postrema (AP). Consequently, no robust evidence indicates that oral leucine intake affects food intake. CVO, circumventricular organ; ME, median eminence.

Several studies have investigated which neuronal circuitries are required for the central effects of leucine (Figure 3). MBH leucine infusion induces the expression of c-Fos, a marker of neuronal activation, in the PVH and ARH as well as in the nucleus of the solitary tract (NTS), which is a structure located in the caudal brainstem [43] (Figure 3). NTS neurons receive sensory information from the gastrointestinal tract and integrate it with other signals to regulate food intake. The reduction in food intake caused by MBH leucine infusion is blocked by either a melanocortin receptor or an oxytocin receptor antagonist, suggesting that a neuronal circuitry between the melanocortin system (POMC cells) and PVH oxytocin-reactive neurons is likely required for the central effects of leucine [43]. The direct administration of leucine into the NTS also reduces food intake and body weight, indicating that both extra-hypothalamic (NTS) and hypothalamic (ARH and PVH) sites are involved in the central effects of leucine on feeding [44] (Figure 3). However, other studies have shown that oral leucine administration does not induce c-Fos expression in the PVH, ARH or NTS [31,45]. In one such study, oral leucine administration induced c-Fos expression in the area postrema (AP), which is an important brain structure responsible for detecting toxins and controlling nausea and vomiting [31] (Figure 3). The activation of cells in the AP by leucine may explain why some studies observed taste aversion in animals consuming a leucine-rich diet [17,46]. Furthermore, orexin-expressing neurons in the lateral hypothalamic area (LHA) are also involved in energy balance regulation and are responsive to amino acids. However, although nonessential amino acids activate orexin-expressing neurons, leucine produces no effects [45] (Figure 3).

## 5. Does Leucine Regulate Food Intake?

As previously mentioned, several studies found that central leucine infusion reduces food intake in rodents [17,40,43,44,46,47]. However, the capacity of leucine to modulate food intake is controversial. Many supplementation approaches have been used to study the effects of leucine on food intake, including leucine supplementation in the drinking water, in the diet and through gavage, as well as by subcutaneous (sc), intraperitoneal (ip), and central injections. To clarify whether leucine supplementation is able to influence feeding behavior, we summarized the results from studies that recorded food intake in leucine-supplemented rodents or humans (Table 1, Table 2 and Table 3). These studies were organized according to the route of leucine supplementation (by central administration, in the diet, or in the drinking water/by other routes). Interestingly, all of the studies evaluating the effects of central leucine infusion observed decreased food intake (Table 1) [17,40,43,44,46,47]. These findings demonstrated that leucine could inhibit food intake by directly affecting the CNS (Figure 3). This result is not surprising because it is well known that the brain is able to sense changing nutrient levels to regulate the energy balance [43]. However, of 30 studies investigating the effects of leucine supplementation in the diet, two studies found increased food intake in leucine-treated animals [48,49], and only four studies reported decreased food intake in the leucine-supplemented groups [17,46,50,51] (Table 2). From these studies, two observed increased taste aversion to the leucine-rich diet, which may explain the reduction in food intake [17,46]. To avoid possible aversive behavior in relation to the diet, several studies supplemented leucine in the drinking water (Table 3). Of 13 studies, two observed that leucine supplementation decreased food intake in specific conditions [52,53]. For example, leucine supplementation in the drinking water decreased the food intake of a polygenic model predisposed to type 2 diabetes (RCS10 mice) but did not affect the food intake of a monogenic model predisposed to obesity and severe insulin resistance (yellow agouti mice) [52]. In another study, leucine supplementation in the drinking water decreased the food intake in males but not in females consuming a regular rodent diet. No effect was observed in mice consuming an HFD [53]. Additionally, one study reported increased food intake in animals supplemented with leucine in the drinking water [54]. No changes in food intake were observed in mice that received leucine supplementation through gavage, ip or sc injections (Table 3). Thus, the central anorexigenic effect of leucine is not well recapitulated when leucine supplementation is provided through an oral form (Figure 3).

**Table 1:** Summary of the studies that investigated the effects of central leucine treatment on feeding.

Reference	Route	Duration	Comments	Effects on Feeding
[40]	icv	Acute	-	**Decreased**
[17]	icv	Acute	-	**Decreased**
[43]	MBH	Acute/7 days	Food intake decreased in the first 2 days	**Decreased**
[44]	NTS	Acute	-	**Decreased**
[46]	icv	Acute	-	**Decreased**
[47]	icv	Acute	-	**Decreased**

icv, intracerebroventricular; MBH, mediobasal hypothalamus; NTS, nucleus of the solitary tract.

**Table 2:** Summary of the studies that investigated the effects of leucine supplementation in the diet on feeding.

Reference	Route	Duration	Comments	Effects on Feeding
[55]	Diet	14 days	Normal and tumor-bearing pregnant rats	No changes
[56]	Diet	Acute	Overnight food-deprived adult and old rats	No changes
[57]	Diet	20 days	Normal and tumor-bearing pregnant rats	No changes
[58]	Diet	12 days	Young and tumor-bearing pregnant rats	No changes
[59]	Diet	10 days	Adult and old rats	No changes
[60]	Diet	14 days	Leucine increased nocturnal meal size	No changes
[61]	Diet	9 weeks	Leucine + phenylalanine supplementation	No changes
[62]	Diet	7 days	-	No changes
[17]	Diet	3 weeks	Aversive behavior to leucine-rich diet in the 1°, but not in the 2° and 21° days.	**Decreased**
[63]	Diet	12 weeks	Healthy elderly men. Energy intake and macronutrient composition were calculated from dietary intake records.	No changes
[64]	Diet	8 weeks	Regular and high-fat diets	No changes
[65]	Diet	21 days	Lactating rats	No changes
[66]	Diet	5 weeks	-	No changes
[67]	Diet	24 weeks	Elderly type 2 diabetic men; 3 days’ dietary intake records to evaluate energy and macronutrient intake.	No changes
[68]	Diet	6 weeks	Previously obese rats	No changes
[21]	Diet	6 weeks	Regular and high-fat diets	No changes
[50]	Diet	7 days	HFD-fed mice; leucine produced similar effects as alanine supplementation.	**Decreased**
[51]	Diet	20 weeks	Mice consuming an HFD	**Decreased**
[69]	Diet	40 days	Old rats recovering from unilateral hind-limb casting	No changes
[70]	Diet	9 months	Aging rats	No changes
[48]	Diet	6 months	Increased food intake only in the first 2 weeks of supplementation	**Increased**/No changes
[71]	Diet	8 weeks	Rats consuming an HFD	No changes
[46]	Diet	4 days	Pronounced taste aversion	**Decreased**
[49]	Diet	24 weeks	Leucine increased food intake only in some points along the experiment	**Increased**/No changes
[72]	Diet	2 weeks	Nutritional recovery	No changes
[73]	Diet	40 days	Adult rats recovering from unilateral hind-limb casting	No changes
[74]	Diet	6 weeks	30% calorie-restricted diet	No changes
[75]	Diet	27 weeks	-	No changes
[47]	Diet	12 days	-	No changes
[76]	Diet	8 weeks	Non-obese, insulin-resistant rats	No changes

**Table 3:** Summary of the studies that investigated the effects of leucine supplementation in the drinking water or by other routes of feeding.

Reference	Route	Duration	Comments	Effects on Feeding
[27]	Water	12 days	Leucine or norleucine supplementation	No changes
[54]	Water	14 weeks	Increased in chow diet group. No change in HFD group.	**Increased**/No changes
[77]	Water	14 weeks	Mice consuming an HFD	No changes
[52]	Water	8 weeks	Food intake decreased in RCS10 mice, but no changes were observed in yellow agouti mice.	**Decreased**/No changes
[78]	Water	8 weeks	Mice consuming an HFD	No changes
[79]	Water	10 weeks	Offspring from HFD-fed mothers	No changes
[80]	Water	8 weeks	Supplementation in normal and high-fat diets	No changes
[81]	Water	17 weeks	Mice consuming normal and high-fat diets	No changes
[53]	Water	9 weeks	Food intake decreased in males, but not females. No leucine effect in mice fed an HFD.	**Decreased**/No changes
[46]	Water	18 days	-	No changes
[31]	Water	6 weeks	Mice consuming normal and high-fat diets and *ob/ob* mice	No changes
[38]	Water	6 weeks	Rats consuming normal and high-fat diets	No changes
[82]	Water	21 weeks	Previously obese mice	No changes
[46]	Gavage	3 days	-	No changes
[31]	Gavage	2 days	-	No changes
[83]	Gavage	10 days	Supplementation during skeletal muscle recovery	No changes
[46]	ip	3 days	-	No changes
[46]	sc	3 days	-	No changes

ip, intraperitoneal; sc, subcutaneous.

The divergent results caused by oral or central leucine supplementation may be explained by the capacity of leucine to cross the blood-brain barrier (BBB) and reach the CNS. Although a previous study demonstrated that a 4% leucine-enriched meal could increase the leucine concentration in the cerebrospinal fluid to 44% [43], whether this change is robust enough to reduce food intake or to persist in the long term remains unknown. In this same study, a high-protein diet did not significantly increase the leucine concentration in the cerebrospinal fluid. Additionally, the changes in leucine levels were approximately seven-fold higher in the plasma than in the cerebrospinal fluid [43]. Therefore, additional studies are warranted to establish the minimum increase in central leucine levels to significantly affect food intake. The BBB and glial cells maintain amino acid concentrations in the CNS parenchyma at well-controlled levels. This is important because several ubiquitous neurotransmitters are amino acids (*i.e.*, glutamate and glycine) or molecules derived from amino acids (*i.e.*, GABA, dopamine, noradrenaline, serotonin, and histamine). Earlier studies have suggested that leucine availability affects the synthesis of amino acid neurotransmitters such as glutamate [34,84]. Abrupt changes in amino acid levels in the brain can cause cell death and serious neuronal dysfunction. For example, excessive activation of the N-methyl-D-aspartate (NMDA) receptor, which is a glutamate receptor, causes neuronal death [85]. Similarly, treatment with monosodium glutamate at a young age, when the BBB is not completely formed, causes lesions in several brain areas [86]. Therefore, the ingestion of specific amino acids does not necessarily lead to pronounced changes in their levels in the brain. Likewise, the direct infusion of amino acids into the brain does not necessarily reproduce the physiological effects caused by oral supplementation and therefore may produce supraphysiological effects. Even brain nuclei that are located near circumventricular organs (CVOs) are not free from the BBB influence. For example, the ARH is close to the median eminence (ME), and the NTS is close to the AP (Figure 3). Blood vessels in these areas allow nutrients and hormones to more easily gain access to the CNS. Nonetheless, the BBB and glial cells in these areas remain capable of controlling abrupt changes in the concentration of molecules. Therefore, the available evidence suggests that oral leucine supplementation produces no or very mild effects on food intake (Figure 3). Because previous studies have suggested a role of leucine supplementation in the treatment of obesity [87], the practical implication is that if leucine regulates the energy balance and favors a reduced adiposity, this effect is likely not mediated by changes in food intake.

## 6. The Effects of Leucine on Body Composition, Obesity, and Energy Expenditure

Despite the lack of evidence indicating that oral leucine intake affects food intake (Table 2 and Table 3), numerous studies have found that leucine supplementation reduces adiposity in specific conditions. For example, leucine supplementation increases body fat loss during a food restriction period [88], decreases the accumulation of fat during aging [70], and partially prevents the development of diet-induced obesity [17,51,54,71,78,80,81]. Nonetheless, notably, not all of the studies have found that leucine prevents diet- or age-induced obesity [31,48,77]. The likely weight-reducing effect of leucine is mediated by changes in energy efficiency caused by increased energy expenditure. For example, leucine supplementation increases energy expenditure in mice consuming an HFD [51,54,81] and in genetically obese mice [52]. She *et al.* [32] provided elegant results indicating that elevated plasma BCAA levels, caused by disruption of the *BCATm* gene, led to increased oxygen consumption. Consequently, *BCATm*^−/−^ mice show decreased body weight and adiposity, although these mice also present elevated food intake. Interestingly, changes in locomotor activity, uncoupling protein levels, sympathetic activity, or thyroid hormone levels were not responsible for the increased energy expenditure of *BCATm*^−/−^ mice. However, the authors observed an active cycle of increased protein synthesis and degradation. This futile cycle consumes energy, which explains the lean phenotype of *BCATm*^−/−^ mice [32,89]. Therefore, the effects of leucine on energy balance regulation may be primarily caused by the stimulation of protein synthesis, thus highlighting that the peripheral effects of leucine likely prevail over its central action to modifying the energy balance. Moreover, the increased protein synthesis caused by high leucine levels does not necessarily lead to higher protein accretion because the rates of protein synthesis and degradation are finely coupled. This fact is clinically relevant because leucine has been studied as a therapeutic supplement to prevent lean mass and protein loss during aging. Therefore, despite the well-known stimulatory effects of leucine on protein synthesis, leucine supplementation in aging rats [48,70,90] or humans [63,67] apparently did not lead to an increase in lean and protein body mass.

Intriguingly, several studies found no effects on adiposity [52,81,82] or even a predisposition to accumulate more body fat [38,48,68] in animals that began to receive leucine supplementation when they were already obese. A likely explanation for these divergent findings is that mTORC1 activation may promote fat storage in adipocytes by suppressing lipolysis and stimulating *de novo* lipogenesis [91]. Additionally, mice with disrupted mTORC1 complex exhibit less adipose tissue, suggesting that fat deposition may depend on mTORC1 activity [92]. BCAA supplementation in pregnant rats consuming a protein-restricted diet restored the fat mass of their offspring to levels similar to those of non-restricted animals [93]. Therefore, the weight-reducing effects of leucine supplementation may be counteracted with the direct action of leucine on adipocytes by stimulating adipogenic processes [91,94]. This effect is observed in situations in which the animals are already obese and insulin resistant before receiving leucine supplementation. Additional studies are necessary to determine the molecular mechanisms responsible for these effects. Some authors have suggested a synergism between BCAA and lipids in the development of metabolic dysfunction in multiple tissues [95,96]. It has been proposed that the increased oxidation of BCAA and fatty acids may lead to mitochondrial stress caused by the abundance of metabolites in the Krebs cycle, which leads to metabolic dysfunctions [95,96].

Leptin is a key hormone involved in the regulation of food intake, energy balance, and glucose homeostasis [39]. Because leucine has direct effects on adipocytes and because leptin synthesis is rapamycin-sensitive, some authors investigated whether leucine could regulate plasma leptin concentrations [97]. These authors observed that increased leucine levels after a meal are partially responsible for inducing the postprandial increase in plasma leptin [97]. If leucine supplementation could increase leptin secretion, this effect may favor weight loss. Nonetheless, obese individuals frequently exhibit hyperleptinemia and leptin resistance [39,98]. Therefore, leucine-mediated leptin secretion may have lower physiological importance in obese individuals. Additionally, obese individuals or rodents already exhibit high circulating BCAA levels [99]. Consequently, leucine/BCAA supplementation may cause lower proportional changes in circulating amino acid levels in obese subjects. Altogether, leucine is unlikely to show beneficial effects as a dietary supplement to help in the treatment of obesity.

## 7. Regulation of Glucose Homeostasis by Leucine

Previous studies observed improved glucose tolerance in leucine-treated animals [51,54,71,78,80,81,82]. For example, leucine supplementation in the drinking water prevented HFD-induced hyperglycemia and insulin resistance in mice [54]. Although a reduced fat mass may explain a portion of these effects, Eller *et al.* [71] observed that the improved glucose control occurred independently of changes in body composition. Therefore, leucine supplementation may improve glucose homeostasis and prevent at least part of diet-induced insulin resistance. The underlying mechanisms involved in the effects of leucine on glucose control remain unknown. The pancreas is a potentially important leucine-target tissue that might affect glucose homeostasis. Leucine stimulates protein synthesis in pancreatic β and acinar cells through the mTOR signaling pathway [9,100]. Additionally, leucine presents insulinotropic properties [61,81,101,102,103]. Therefore, increased insulin secretion in leucine-supplemented individuals could improve their postprandial glucose levels. However, some caution when using leucine supplementation in specific situations is recommended. Leucine supplementation in pregnant rats resulted in decreased β-cell formation in their offspring, which could potentially increase the risk of type 2 diabetes mellitus later in life [104]. Additionally, leucine supplementation enhances tumor growth in a murine model of pancreatic cancer [75].

Other mechanisms, in addition to the regulation of insulin secretion, are likely to be involved in the effects of leucine on glucose control. Several studies have observed that leucine-treated animals exhibited improved insulin sensitivity [54,71,80,81]. Additionally, leucine supplementation decreases glucose-6-phosphatase expression in the livers of mice consuming an HFD, suggesting reduced gluconeogenesis [49,54]. Leucine supplementation increases SIRT1 expression and prevents mitochondrial dysfunction in the livers of diet-induced obese mice [80]. Furthermore, hepatic steatosis and lipid metabolism were improved in leucine-supplemented animals [50,51,54,78,80,105]. BCAA or leucine supplementation also affects glucose metabolism and glycogen synthesis in skeletal muscle [71,78,80,106]. Therefore, the direct effects of leucine on insulin-sensitive tissues such as the liver and skeletal muscle may influence whole-body glucose homeostasis. Paradoxically, classical studies have found that high physiological BCAA concentrations inhibit the early steps in insulin signaling [4] and that amino acid infusion acutely causes insulin resistance in human skeletal muscle [107]. More recently, some studies revealed that obese and lean humans differ in terms of BCAA metabolism and that BCAA may contribute to insulin resistance in obesity in humans [95,96]. Balage *et al.* [66] observed that five-week leucine supplementation induced a delay in postprandial stimulation in the early steps of muscle insulin signaling, leading to overall glucose intolerance. Insulin resistance is also induced by mTOR/S6K1 pathway overactivation [108]. Accordingly, the absence of S6K1 protects against age- and diet-induced obesity and enhances insulin sensitivity [15]. Additionally, hyperinsulinemia leads to insulin resistance in the liver and skeletal muscle through a rapamycin-sensitive mechanism [109]. Therefore, excessive mTOR/S6K1 activation by either amino acids or insulin leads to insulin resistance. The mechanism proposed to explain these effects is increased S6K1-mediated phosphorylation of the serine residues of insulin receptor substrate (IRS)-1 [109,110,111] (Figure 1). Markedly elevated S6K1 activity and increased serine phosphorylation of IRS-1 are observed in animals consuming an HFD or in genetically obese and diabetic models [15,112,113,114]. Therefore, serine phosphorylation of IRS-1 is considered to be a key feature in insulin resistance.

Because adipose tissue is an important leucine-responsive tissue [28], leucine supplementation may change the secretion pattern of adipokines to a more favorable profile. Previous studies have found that leucine supplementation in previously obese rats increased adiponectin levels [68]. In another study, leucine supplementation reduced inflammatory marker levels in white adipose tissue [78]. Accordingly, a combined leucine and pyridoxine supplementation increased adiponectin levels and reduced the concentrations of oxidative and inflammatory markers in the plasma of obese subjects [115]. Therefore, the improved glucose control in leucine-supplemented animals may be secondary to a more favorable inflammatory profile and cytokine secretion pattern in the adipose tissue. Additionally, increased fat mass due to leucine supplementation may increase glucose uptake by adipocytes, which may help to reduce blood glucose levels in obese animal models [49,82].

The CNS is critically involved in the regulation of glucose homeostasis [116]. Although no clear evidence indicates that oral leucine supplementation is able to affect food intake, the effects of leucine in glucose control may require lower variations in central leucine levels that can be achieved through oral supplementation. Different thresholds that affect food intake and glucose control have been reported in other situations. For example, a low-dose leptin treatment that did not affect the food intake or body weight of leptin-deficient (*ob/ob*) mice was able to reduce their glucose and insulin levels [117]. Previous studies have observed that leucine depolarizes POMC neurons *in vitro* [43] and that icv leucine infusion increases hypothalamic POMC expression [17]. Because POMC neurons are critically involved in the regulation of glucose homeostasis and hepatic insulin sensitivity [118], the activation of POMC neurons may be responsible for mediating at least a portion of the anti-diabetic effects of leucine.

Finally, scarce information is available concerning the effects of leucine supplementation on the secretion of gut hormones or on the gut microbiota. Changes in the gut microbiota [119] or gut hormones [120] may produce significant effects in energy and glucose homeostasis regulation. One of the few studies that investigated the effects of leucine on the gut observed that leucine stimulates glucagon-like peptide-1 (GLP-1) mRNA levels and secretion [121]. Either GLP-1 or exendin-4, a long-acting GLP-1 agonist, has been shown to enhance glucose tolerance [122,123]. Nonetheless, future studies that directly evaluate these possibilities are required to enhance our understanding of the underlying mechanisms involved in the potential beneficial effects of leucine on glucose homeostasis.

## 8. Concluding Remarks

**Figure 4:**
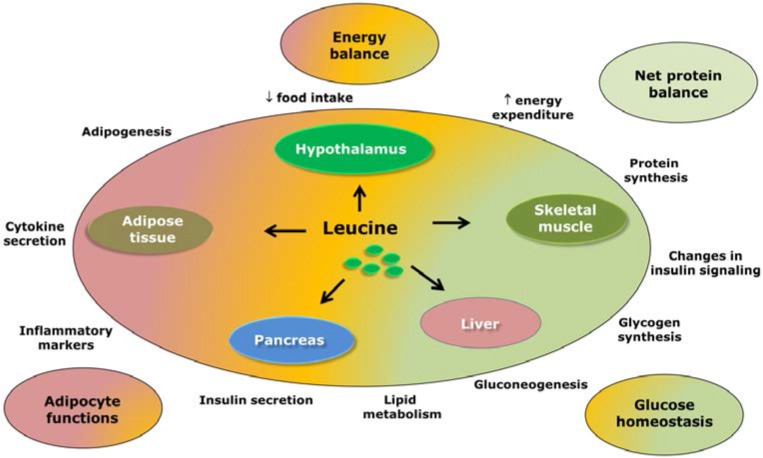
Possible effects of leucine supplementation in the regulation of energy balance and glucose homeostasis. This scheme summarizes the available evidence regarding the likely effects of leucine supplementation in different tissues and its subsequent consequences.

The potential effects of leucine supplementation are summarized in Figure 4. Because leucine regulates several cellular processes via mTOR and possibly through other signaling pathways, the likely beneficial effects of leucine supplementation have been evaluated in a variety of situations, including as a dietary supplement for the treatment of obesity and diabetes mellitus.

Part of the interest in studying the potential therapeutic application of leucine supplementation emerged from studies observing that leucine could be one of the “active ingredients” in high-protein diets [17,51,87,124]. Several studies have found that high-protein diets may be beneficial for weight management and for controlling glucose levels [87,124,125]. However, in the present review, we focused only on studies that directly investigated leucine/BCAA supplementation. Among the practical implications that emerged from the data summarized and discussed in the present review, the first is that no robust evidence indicates that oral leucine supplementation can reduce food intake. Notably, central leucine injection appears to decrease food intake; however, this effect is not well reproduced when leucine is provided as a dietary supplement, which calls its therapeutic application into question. Additionally, several studies indicated that leucine supplementation may help to decrease body adiposity in specific conditions. However, additional studies are warranted to assess the effects of leucine supplementation in already-obese subjects. The studies that initiated leucine supplementation in already-obese animals found no beneficial effects of leucine or even found worsening of the degree of adiposity. Therefore, based on the presented data, leucine supplementation is not likely to be helpful as a dietary supplement for treating obesity. Finally, we discussed the potential therapeutic effects of leucine in improving glucose homeostasis. Although some studies have found that leucine supplementation improves glucose tolerance, the underlying mechanisms involved in these effects remain unknown and may be partially dependent on weight loss.
